# Development of a Peptide ELISA for the Diagnosis of Aleutian Mink Disease

**DOI:** 10.1371/journal.pone.0165793

**Published:** 2016-11-01

**Authors:** Fanshu Ma, Lei Zhang, Yang Wang, Rongguang Lu, Bo Hu, Shuang Lv, Xianghong Xue, Xintong Li, Mingyu Ling, Sining Fan, Hailing Zhang, Xijun Yan

**Affiliations:** Division of Infectious Diseases of Special Animal, Institute of Special Animal and Plant Sciences, The Chinese Academy of Agricultural Sciences, Changchun, China; Ella Foundation, INDIA

## Abstract

Aleutian disease (AD) is a common immunosuppressive disease in mink farms world-wide. Since the 1980s, counterimmunoelectrophoresis (CIEP) has been the main detection method for infection with the Aleutian Mink Disease Virus (AMDV). In this study, six peptides derived from the AMDV structural protein VP2 were designed, synthesized, and used as ELISA antigens to detect anti-AMDV antibodies in the sera of infected minks. Serum samples were collected from 764 minks in farms from five different provinces, and analyzed by both CIEP (a gold standard) and peptide ELISA. A peptide designated P1 (415 aa–433 aa) exhibited good antigenicity. A novel ELISA was developed using ovalbumin-linked peptide P1 to detect anti-AMDV antibodies in mink sera. The sensitivity and specificity of the peptide ELISA was 98.0% and 97.5%, respectively. Moreover, the ELISA also detected 342 early-stage infected samples (negative by CIEP and positive by PCR), of which 43.6% (149/342) were true positives. These results showed that the peptide ELISA had better sensitivity compared with CIEP, and therefore could be preferable over CIEP for detecting anti-AMDV antibodies in serological screening.

## Introduction

Aleutian disease (AD), a common infectious disease worldwide, is caused by Aleutian Mink Disease Virus (AMDV) [1]. The virus is a member of the genus *Amdoparvovirus*, subfamily *Parvovirinae*, family *Parvoviridae*, and is distant to other parvoviruses [2]. AMDV is a single-stranded DNA virus with a full-length genome of 4.8 kb, encoding two structural proteins VP1 and VP2, and three non-structural proteins NS1, NS2 and NS3 [3]. Clinical manifestation of AD varies from a mild non-persistent disease to a persistent, potentially fatal disease. The main symptoms of typical AD are anemia, cachexia, polydipsia, reduced reproductive abilities, hypergammaglobulinemia, renal failure and increased susceptibility to bacterial infections. A consequence of the disease is the deposition of antigen-antibody complexes in tissues of different organs which can cause conditions such as nephritis [4,5]. Also, a decline in the reproductive ability and the resulting low-quality fur of infected animals can result in huge economic losses to farmers and the fur industry.

AMDV can infect several members of the *Mustelidae* family, of which mink and ferrets are common hosts. Raccoons, otters, raccoon dogs, and striped skunks have also been reported to be infected [6–10]. Humans are thought to be susceptible for AMDV infection but the infections are limited to the workers who come in close contact with minks [11].

AMDV has been found to be highly resistant to disinfectants and high temperatures. Infected tissues have been reported to remain infectious even after heating at 80°C for 30 min or 99.5°C for 3 min [12]. Moreover, AMDV-contaminated manure can only be effectively inactivated at 65°C for at least 3–4 days [13]. The transmission occurs horizontally by direct and indirect contact, or vertically to mink kits [14,15]. Therefore, it is difficult to sanitize an infected farm. To date, there is neither an ideal vaccine nor effective treatment strategies against AMDV infections. The only effective control is based on continual serological screening by counterimmunoelectrophoresis (CIEP) followed by elimination of seropositive animals [16].

As the gold standard, CIEP is routinely used for the detection of AMDV. CIEP was developed in the 1970s using an organ-produced antigen. In the 1980s, *in vitro* cultured (Crandell feline kidney cells) antigen for AMDV-G strain was widely utilized [17]. Although CIEP is highly specific, it has poor sensitivity, is time-consuming and requires large quantities of antigen. Furthermore, the results are subjective, and require experience, leading to false-positive readings. Other immunoelectrophoretic assays, such as indirect counter-current electrophoresis, counter-current line absorption immunoelectrophoresis [18,19] and additive CIEP [20] have been developed, however, these methods have not been used in field testing for various reasons. PCR methods amplifying the *VP2* or the *NS1* gene have also been used to diagnose AMDV infections. These methods are more sensitive than CIEP in the early-stage of infection. However, due to the transient nature of viremia in most of infected minks, PCR can only be used during a limited window post-infection, making it a supplementary test to diagnose AD [21–23], whereas antibodies can be detected for 24 weeks or more.

Enzyme-linked immunosorbent assay (ELISA) are the most common methods for routine screening of AD. An ELISA based on baculovirus-expressed AMDV VP2 capsid has been developed, and this test has sensitivity and specificity of 99% and 97%, respectively, compared to CIEP [24,25]. However, this process requires large quantities of VP2 protein that is obtained by propagation and purification of recombinant baculovirus. An alternative would be to use synthetic peptides, which have been applied for the diagnosis of other diseases [26–32]. A detection method utilizing peptides for rapid screening of anti-AMDV antibodies has not yet been developed.

In this study, we used bioinformatics to map the linear B-cell epitopes of AMDV VP2 capsid protein, and we identified six peptides from highly conserved regions with high levels of predicted antigenicity. These six peptides, corresponding to amino acids 288–309, 325–340, 415–433, 514–532, 556–567, and 604–616, were synthesized, and a novel indirect ELISA was developed for the detection of anti-AMDV antibodies in infected mink sera. Sensitivity (Se) and specificity (Sp) were evaluated by comparing the results of the novel ELISA with that of CIEP.

## Material and Methods

### Ethics statement

All animals were handled humanely according to the rules described by the Animal Ethics Procedures and Guidelines of the People’s Republic of China. Sera used in this study were collected from eight private mink farms in China, with oral consent from the farm owners. Blood was obtained using toe-nail clips, and no animals were sacrificed for the sake of this study.

### Blood samples

A total of 1106 blood samples were collected from minks with varied age and color types from eight farms located in five different provinces in China. Blood was obtained using toe-nail clips. After centrifugation at 664×g for 10 minutes, the sera were stored at −20°C. Negative and positive sera, which were tested both by CIEP and PCR, were obtained from specific-pathogen-free animals from commercial a source (JL TEYAN Biological Technology LLC, China).

### B-cell Epitope Prediction and peptide synthesis

The online tools Immune Epitope Database Analysis Resource (IEDB) [33, 34] and ABCpred [35] were used to predict the B cell linear epitope regions of the VP2 sequence. The Kolaskar and Tongaonkar method, which has an accuracy of 75% [35], was used for IEDB-based epitope prediction. DNAstar and BcePred [36] were used to analyze the physicochemical properties such as hydrophilicity, polarity, flexibility, and exposed surface [37–40]. SWISS-MODEL software was used to predict the 3D structure of the VP2 protein [41]. Based on our previous observations on the molecular epidemiology of AMDV in China [42], we focused on conserved regions of the VP2 protein. ABCpred server selected 16 amino acid residues as a unit using neural networks. Through the above comprehensive analysis (B cell linear epitope regions, 3D structure and conserved regions of VP2 protein), the peptides with highest predicted antigenicity conservation were synthesized by Sangon Biological Engineering Technology (Shanghai, China). According to the physicochemical properties and 3D structure, a Cys was added to the peptide N- or C-terminus and conjugated with an ovalbumin (OVA). Since OVA is considerably larger than each individual peptide, Cys was added to the ends to minimize the influence of OVA folding on the peptide function. Each OVA-conjugated peptide was purified to (≥95% purity by solid-phase extraction, followed by reverse-phase high pressure liquid chromatography.

The antigenicity of peptides was analyzed by indirect ELISA. The peptides were used as antigen and the ELISA was performed using commercially available negative and positive sera. The P/N values were analyzed using one-way analysis of variance (ANOVA) using SAS 9.0. Duncan’s multiple range tests were used to determine statistical significance between peptides. The peptide with good antigenicity was selected for development of the peptide ELISA method.

### AMDV Peptide ELISA

OVA-conjugated peptide was diluted to 10, 5, 2.5, 1.25, 0.6, 0.3, 0.15, 0.07 μg/mL. Positive and negative sera were diluted to 1:50, 1:100, 1:200, 1:400, and horseradish peroxidase-conjugated goat anti-cat IgG (KPL) was diluted to 1:1000, 1:2000, 1:4000, 1:8000, 1:16000, 1:32000. Checkerboard titration was performed to determine the optimal dilutions of coating peptide, sera and goat anti-cat IgG/HRP. The blocking buffer was optimized form 1% Bovine Serum Albumin (BSA, KPL), 1% pig gelatin (Sigma), 1% fish gelatin (Sigma), 1% casein (Sigma), 5% skim milk powder (BD) and 5% goat serum (Gibco).

For ELISA, OVA-conjugated peptides were coated onto 96-well microplates (Corning Costar, U.S.A.) overnight at 4°C with 100 μL 0.5 μg/mL in 50 mM carbonate/bicarbonate buffer, pH 9.6 (Na_2_CO_3_ 1.59g, NaHCO_3_ 2.93g, water up to 1 L). After three washes with the Tris-Buffered Saline and 0.1%Tween-20 (TBST) at pH 8.6 (NaCl 8g, KCl 0.2g, Tris-base 1.21g, tween-20 1mL, water up to 1 L). the plates were blocked with 100 μL/well of blocking buffer (5% skim milk powder in washing buffer) for 2 h at 37°C. The serum samples diluted to 1:200 in dilution buffer were then added (100 μL/well), and incubated for 1 h at room temperature (RT). Following five washes with washing buffer, 100 μL/well goat anti-cat IgG/HRP diluted 1:4000 in TBST was added, and the plates were incubated for 1 h at RT. After washing five times, 100 μL of tetramethylbenzidine (TMB) substrate (Sigma-Aldrich) was added to each well, and the plates were incubated for 10 min at RT before the reaction was stopped with 1 M HCl (100 μL/well). The absorbance was measured at 450 nm. The mean OD of two blank wells was subtracted from each OD value of the sample. Each plate contained positive and negative controls.

### Counterimmunoelectrophoresis (CIEP)

CIEP was performed with commercially available antigen (JL TEYAN Biological Technology Limited liability company, China) and following the method provided by the manufacturer. Briefly, 6 cm × 6 cm glass plates were coated with 20 mL of 1% agarose gel in barbital buffer, pH 8.6 (barbital 1.84 g, barbital sodium 10.3 g, water up to 1 L), and the gel was punched using capillary tips. The serum samples were added in anodal wells, and the antigen was added in cathodal wells. Each plate contained a positive control. The samples were subjected to electrophoresis for 45 min at 90 volts, and the results were read by two experienced observers.

### PCR detection

This method was based on the study conducted by Jensen et al [43], and the following primers were used: forward, 5′ CAT ATT CAC TGT TGC TTA GGT TA 3′; reverse, 5′ CGT TCT TTG TTA GTT AGG TTG TC 3′. The amplification mixture was initially incubated at 94°C for 5 min, and then 45 cycles of denaturation at 94°C for 30 s, annealing at 55°C for 30 s, elongation at 72°C for 30 s and a final elongation at 72°C for 7 min. The PCR product of 374 bp was visualized by agarose gel electrophoresis and UV transillumination.

### Statistical analysis

The cut-off value was determined by Receiver Operating Characteristic (ROC) curve. Based on the detection results of 377 random sera obtained by the CIEP and ELISA methods, ROC curves and area under the curve were plotted, and the ELISA sensitivity (Se) and specificity (Sp) were calculated. According to the Youden index (Youden = Se + Sp– 1), a cut-off value for ELISA was determined. OD 450 greater than the cut-off value was judged to be positive, and less than the cut-off value as negative.

To detect early-stage infections, samples which were antibody-negative by CIEP and genome-positive by PCR were selected for testing with peptide ELISA.

The coefficient of variation (CV) was obtained by intra-plate repeatability and inter-plate repeatability. To evaluate intra-plate repeatability, four replicates of 24 samples were tested. Inter-plate repeatability was evaluated by using the same samples in four different plates. CV was calculated as CV = (standard deviation / mean) × 100%

Agreement within CIEP and peptide ELISA data was obtained by analysis of the same serum. The following diagnostic indicators were deduced by the given formulae:

Coincidence rate=(true positive sample number + true negative sample number)/number of total samples

Sensitivity (SE)=number of true positive samples/(true positive sample number + number of false negative samples)

Specificity (SP)=number of true negative samples/(number of true negative samples + number of false positive samples).

## Results

### Detection with CIEP and PCR

A total of 1106 blood samples were included in this study. 764 samples were collected in 2014, of which 502 were CIEP-positive and 262 were CIEP-negative. In 2015, 342 samples in the early stage of infection were collected and tested by CIEP and PCR from minks aged 45–60 days. Details of the samples are shown in Table 1. All these samples were anti-AMDV antibody-negative by CIEP and AMDV genome-positive by PCR.

**Table 1:** Sampling data for each mink herd.

Herd	CIEP positive sera in 2014 (n)	CIEP negative sera in 2014 (n)	Early infection sera in 2015 (n)	Total
**Province 1**	240	19	23	282
**Province 2**	174	54	239	467
**Province 3**	88	23	80	191
**Province 4**	0	34	0	34
**Province 5**	0	132	0	132
**Total**	502	262	342	1106

### Prediction of the B-cell epitopes

The physicochemical properties of amino acids (aa) within a protein, especially hydrophilicity, flexibility and surface probability, are important for the prediction of B-cell epitopes [34, 37, 38]. Some flexible regions, such as turns and random coils, provide powerful evidence for epitope recognition [39]. We have earlier observed that the frequency of VP2 protein mutation in AMDV was higher than that of both Aleutian ferret disease virus (AFDV) and mink enteritis parvovirus (MEV) [16], based on which, we compared the sequences of VP2 proteins and were able to identify and exclude the hypervariable regions, such as aa 3–13 and aa 231–242.

**Fig 1:**
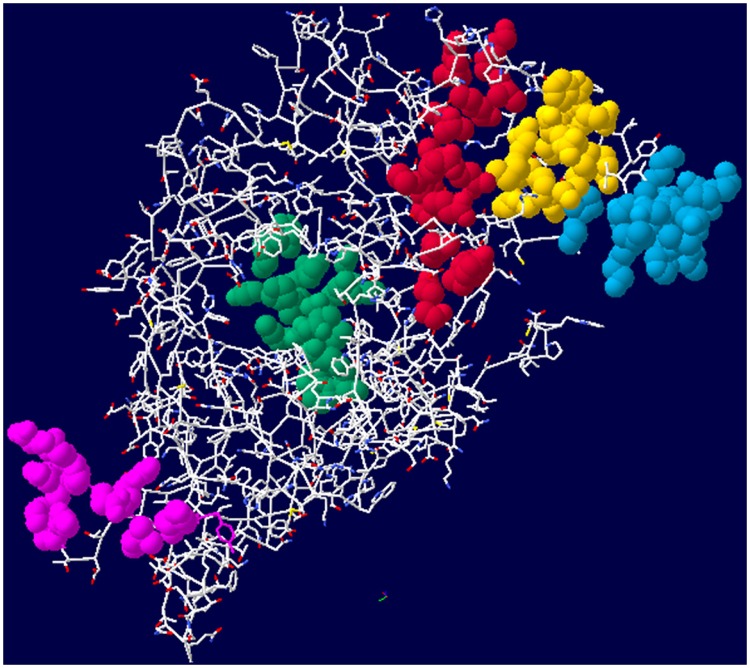
VP2 protein structure model using Swiss-Model server. Five of the six synthesized peptides are labeled in different colors. 288 aa–309 aa (P3) is blue, 325 aa–340 aa (P2) is yellow, 415 aa–433 aa (P1) is red, 514 aa–532 aa (P4) is green, 556 aa–567 aa (P6) is pink.

Analyses showed that AMDV VP2 was 619 aa long (GenBank: KU243336). Examination of the physicochemical properties showed that the isoelectric point (pI) was 5.37, the protein had 78 acidic amino acids, 57 basic amino acids, 208 polar amino acids and 167 hydrophobic amino acids. Overall, the hydrophilic regions of the VP2 protein were distributed throughout the sequence, and the hydrophilic regions were 4 to 28, 196 to 235, 316 to 346, 367 to 401, and 414 to 449. Prediction by DNAstar BcePred showed that amino acids at the positions 4 to 18, 187 to 200, 195 to 241, 291 to 306, 328 to 349, 413 to 439, 516 to 530, and 556 to 569 had higher surface exposure index. PredictProtein analysis results suggested that VP2 protein structure was 1.6% helix, 14.9% strand, and 83.3% loop. The Swiss-Model server generated a structure for VP2 based on amino acid sequence homology with the published crystal structure of the minute virus of mice (PDB id: 1z14, 44.09% of sequence identity) in the Protein Data Bank (PDB). Fig 1 shows the proposed structure model of AMDV VP2. With comprehensive consideration as described above, six peptides from highly conserved regions with high levels of predicted antigenicity were identified and selected as potential epitopes. The structures and relative position of five peptides are shown in Fig 1, and labeled in different colors. The model structure lacks 26 amino acids from 593 to 619, so the peptide aa 604–616 is not shown in this structure.

### Antigenicity of peptides by ELISA analysis

**Fig 2:**
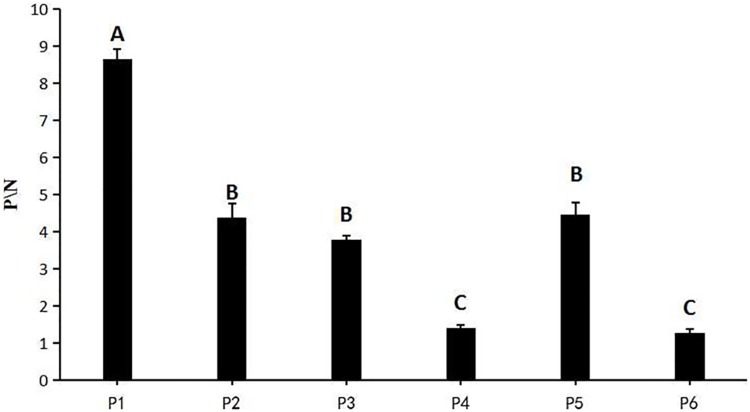
P/N values of all the peptides tested with anti-AMDV positive and negative sera. P1: 415 aa–433 aa, P2: 325 aa–340 aa, P3: 288 aa–309 aa, P4: 514 aa–532 aa, P5: 604 aa–616 aa, P6: 556 aa–567 aa; the uppercase letters indicate significant differences at 0.01 level, the same letters denote no significant difference.

The reactivity of the peptides to the negative and positive sera in ELISA was determined and the results are shown in Fig 2. Five peptides (P1, P2, P3, P5, and P6) were clearly recognized by the anti-AMDV antibodies. Compared with the other peptides, P1 appeared to have the highest reactivity (*P<0*.*01*). After further experimentation, P1 was chosen as the antigen to establish the peptide ELISA.

### Optimization of the peptide ELISA procedure

**Fig 3:**
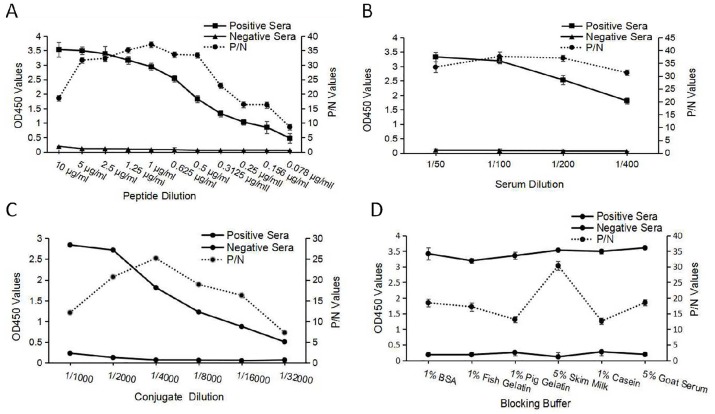
Optimization of peptide ELISA working conditions. (a) Optimization of peptide coating concentration. (b) Serum sample and (c) conjugate (HRP-labeled goat anti-cat IgG) dilutions, (d) optimization of the blocking buffer.

ELISA was optimized using the checkerboard method. According to the OD450 values and P/N values, the ideal peptide coating concentration was set at 0.5 μg/mL (Fig 3a). The optimal dilution of the sera was 1:200 (Fig 3b), and the optimal dilution of secondary goat anti-cat serum was 1:4000 (Fig 3c). The blocking buffer containing 5% skim milk in TBST was found to be better than 1% BSA, 1% pig gelatin, 1% fish gelatin, 1% casein, and 5% goat serum (Fig 3d). During the optimization of peptide concentration, the OD450 declined rapidly from 0.6 μg/mL to 0.3 μg/mL. In order to perform a detailed analysis, a second round of checkerboard ELISA was performed, using peptide concentrations of 1, 0.5, and 0.25 μg/mL (Fig 3a), following which, the optimal peptide concentration for coating was determined to be 0.5 μg/mL.

### Cut-off value for the peptide ELISA

**Fig 4:**
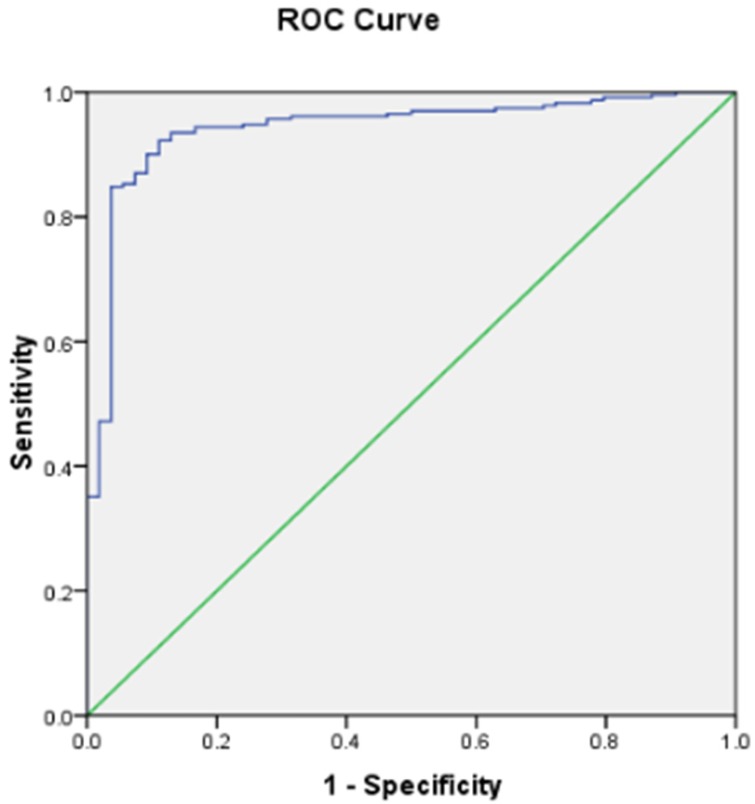
Validation of the peptide ELISA by ROC. The OD450 values of 377 confirmed sera obtained from peptide ELISA were compared with the CIEP results by ROC analysis. The AUC value was 0.948 with a standard error of 0.02.

To obtain the cut-off value of the ELISA, 377 random sera from five major mink farming provinces of China were assayed by ELISA and CIEP; 158 were CIEP-negative and 219 were CIEP-positive. The ROC curve was established by considering CIEP results as the gold standard, cut-off values were determined using the Youden index, and the results are shown in Fig 4. The area under the ROC curve (AUC) was 0.948 with a standard error of 0.02. The highest Youden index was 0.811, and corresponding cut-off value was 0.4183, and using this value, the sensitivity (Se) and specificity (Sp) of peptide ELISA were 0.922 and 0.889, respectively.

### Application of the peptide ELISA to assay the serum samples and comparison with CIEP

Using the ELISA cut-off values, both CIEP and ELISA were used to assay 764 serum samples. The results are shown in Table 2. Amongst these, 502 and 262 samples were found to be CIEP-positive and CIEP-negative; whereas, 498 and 266 samples were ELISA-positive and ELISA-negative, respectively (the ELISA cut-off value was defined to be 0.4183 based on ROC curve). Accordingly, the Se and Sp for the ELISA were calculated to be 98.0% and 97.7%, respectively. The coincidence rate between the two methods was 97.9%.

**Table 2:** Comparison between CIEP and ELISA methods for the detection of AMDV antibodies.

	# of samples detected by CIEP		Total
	Positive	Negative	
**ELISA Positive**	492	6	498
**ELISA Negative**	10	256	266
**Total**	502	262	764
**Sensitivity**	98.0%,		
**Specificity**		97.7%	

### Comparison between CIEP and ELISA methods for the detection of early-stage infection samples

The peptide ELISA was used to detect 342 early-stage infection samples. All of the samples were anti-AMDV antibody-negative by CIEP, and AMDV genome-positive by PCR. The OD values of these samples obtained by ELISA are shown in Table 3. Amongst the 342 early-infection sera, 149 samples were positive and 193 negative by ELISA, and the positivity rate was 43.6%.

**Table 3:** Comparison between CIEP and ELISA methods for the detection of early-stage infection samples.

	# of samples detected by CIEP		Total
	Positive	Negative	
**ELISA Positive**	0	149	149
**ELISA Negative**	0	193	193
**Total**	0	342	342

### Comparison of the sensitivities of ELISA and CIEP

The sensitivities of ELISA and CIEP were evaluated using the 10 AMDV-positive samples that were positive in both CIEP and PCR. In CIEP, samples were serially diluted 2-fold from 1:2 to 1:256, and in ELISA, they were diluted from 1:20 to 1:10,240, and 1:50 to 1:12,800. The assays was performed in triplicate for each dilution. The data are shown in Table 4. Using ELISA, the maximum and minimum dilutions of the antibody that could detect AMDV-positive samples were 1:6400 and 1:40, respectively. By CIEP, the maximum and minimum dilutions were 1:128 and 1:2, respectively. These data indicated that peptide ELISA was more sensitive than CIEP in detecting anti-AMDV antibodies, and may be better for AMDV serological screening.

**Table 4:** Comparison of the sensitivities of ELISA and CIEP.

Method	Sample code									
	1	2	3	4	5	6	7	8	9	10
**ELISA**	1:6400	1:5120	1:1280	1:640	1:640	1:320	1:160	1:80	1:40	1:40
**CIEP**	1:128	1:16	1:64	1:32	1:16	1:16	1:16	1:16	1:8	1:2

### Determination of the specificity of the peptide ELISA

Specificity of the peptide ELISA was evaluated using serum samples positive for mink enteritis virus (MEV) and for canine distemper virus (CDV). The resulting OD value for the MEV serum was 0.272, and for the CDV serum was 0.149, so both of them were negative using the established ELISA cut-off (data not shown). Taken together, these results indicated that other common virus-positive sera of minks had minimal cross-reactivity with peptide P1, and that the peptide ELISA specifically detected anti-AMDV antibodies.

### Repeatability assays

A total of 24 sera were selected to evaluate the assay repeatability. The summary of the results is shown in Table 5. The intra-plate and inter-plate CV of the peptide ELISA was 0.33% to 7.60% and 0.16% to 8.87%, respectively.

**Table 5:** Results for the repeatability assay.

Repeatability assay	Plate	Maximum CV (%)	Minimum CV (%)
**Intra-plate repeatability**	Plate 1	7.60%	0.33%
**Inter-plate repeatability**	Plate a vs Plate b	6.17%	0.16%
	Plate b vs Plate c	8.87%	0.72%
	Plate c vs Plate d	6.39%	0.58%
	Plate d vs Plate a	7.84%	0.37%

CV = coefficient of variation.

## Discussion

The aim of this study was to develop a simple, sensitive and specific ELISA for detecting anti-AMDV antibodies based on OVA-conjugated peptides. We mapped linear B-cell epitopes of the VP2 amino acid sequence using bioinformatics. Based on an earlier observation that different field strains of AMDV have regions of variability in their gene sequences [42], we excluded such regions of VP2. Six potential antigenic peptides of VP2 predicted from bioinformatics analyses were synthesized to be more than 95% purity and subjected to evaluation for diagnostic purposes. The analysis revealed that one peptide (P1: aa 413–449) was hydrophilic, and had higher surface exposure index, consistent with published studies, which have reported that the aa 428–448 region may be associated with antibody-dependent enhancement of infection, virus neutralization and immune complex formation [44–46]. The sequence of aa 428–446 is almost identical to aa 415–433 of our VP2 sequence (AMDV strain in China has a poly-G deletion near the N-terminus). Based on these results, we chose to the 415–433 peptide (P1 peptide), which indeed had the highest reactivity with anti-AMDV antibodies. By using an OVA-linked P1 peptide, we developed a novel ELISA for assaying mink sera. We evaluated the AMDV peptide ELISA for sensitivity and repeatability, and also compared this method with CIEP. The results indicated that the peptide ELISA had higher sensitivity than CIEP, especially for detecting the early infection period.

At present, countries all over the world have directed their attention towards eradicating AD. Every year, a large number of serum samples need to be screened for anti-AMDV antibodies to accomplish this goal, and this number is increasing each year [24, 25]. The CIEP method has been unable to meet the clinical needs. Recently, at least three different ELISA systems have been developed to detect anti-AMDV antibodies. AMDV-G ELISA is based on the AMDV-G that was produced *in vitro*, but this method has a poor sensitivity of 37.6% [25]. The other two methods are VP2 ELISA and VP2332-452-ELISA [24, 47]. VP2 ELISA is employs a baculovirus-expressed whole VP2 protein as antigen. The sensitivity and specificity of this method were 99% and 97% as compared to CIEP, respectively. VP2332-452-ELISA is based on *E*. *coli*-expressed VP2332-452 protein as antigen. This method has a sensitivity of 97.9% and a specificity of 97.3% relative to CIEP. Synthetic peptides have also been applied for the diagnosis of various other diseases [26–32], but ours is the first such application for AMDV. Compared with CIEP, peptide ELISA was more sensitive to detect early-stage of infection, has a simpler methodology, and can be applied for routine screening for the detection of anti-AMDV antibodies.
